# How do patients want to learn of results of clinical trials? A survey of 1431 breast cancer patients

**DOI:** 10.1038/sj.bjc.6604119

**Published:** 2007-12-04

**Authors:** L Johnson, P Barrett-Lee, P Ellis, J M Bliss

**Affiliations:** 1 ICR-CTSU, Section of Clinical Trials, Sir Richard Doll Building, Institute of Cancer Research, Cotswold Road, Sutton, Surrey SM2 5NG, UK; 2 Velindre Cancer Centre, Velindre Hospital, Whitchurch, Cardiff CF14 2TL, UK; 3 Department of Medical Oncology, Thomas Guy House, Guy's Hospital, St Thomas Street, London SE1 9RT, UK

**Keywords:** trial results, participating patients, lay terms

## Abstract

Questionnaires were circulated to UK patients and health care professionals (HCPs) participating in the Taxotere as Adjuvant ChemoTherapy (TACT) trial in autumn 2004 asking if and how trial results, when available, should be conveyed to patients. A total of 1431 (37% of surviving UK TACT patients) returned questionnaires. In all, 30 (2%) patients did not want results. In all, 554 (40%) patients preferred to receive them via their hospital; 664 (47%) preferred results posted directly to their home, 177 (13%) preferred a letter providing a telephone number to request results. Six hundred and twelve patients thought results should come directly from the trials office. One hundred and seventy-six HCPs from 89 UK centres (86%) returned questionnaires. In all, 169 out of 176 patients (96%) thought results should be written in lay terms for patients. Seventy (41%) preferred patients to receive results via their hospital; 64 (38%) preferred a letter providing a telephone number to request results, and 32 (19%) preferred results posted directly to patients. Thirty-one HCPs (18%) thought results to patients should come directly from the trials office. A total of 868 (61%) patients thought next of kin of deceased patients should receive results, 543 (38%) did not; 47 (27%) HCPs thought they should; 118 (68%) did not.

The view that researchers have an obligation (Fernandez *et al*, 2003) to provide results of clinical trials to those who participate in them has reached mainstream thinking, with professional medical bodies (Royal College of Physicians, 1990) supporting this approach. However, within the UK, recently updated national guidelines on information sheets for trial participants (www.nres.npsa.nhs.uk) advise caution in disseminating results to participants, an approach which is justified by the mixed findings from several studies (Snowdon *et al*, 1998; Richards *et al*, 2003; Dixon-Woods *et al*, 2006). The guidelines' distinction between ‘broad scientific results of a trial’ and ‘results of relevance to the individual’ is welcomed, as the broad scientific results may need to be presented to participants in the context of (possibly) differing results from similar trials, which taken individually may be confusing or inconclusive, and normally have no bearing on the patients' future clinical care.

For cancer treatment trials in patients with early disease, a lapse of several years from trial entry to availability of results means that unexpected contact to convey results could cause undue alarm. Two further studies (Partridge *et al*, 2004, 2005) both show that the majority of cancer patients would like to receive results, but provide conflicting data on whether patients want results conveyed by their doctor or they are comfortable receiving them by post. For trials with survival as an endpoint, the issue of how, when and whether to disseminate results to the next of kin of those who have died has not been fully explored.

In this paper, we report an attempt to evaluate whether patients who took part in the Taxotere as Adjuvant ChemoTherapy (TACT) trial (Barrett-Lee *et al*, 2002) wish to be informed of the trial results when they become known. We sought preferences relating to the possible practical mechanisms for distributing them to participating patients, and in the case where the patient has subsequently died, the extent to which participants supported the idea that their next of kin should receive the results. We compared the views of participating patients with those of the health care professionals involved with the TACT trial, that is those most likely to convey trial results to patients, who will appreciate the scientific context, and practical considerations which may not be obvious to patients.

Taxotere as Adjuvant ChemoTherapy is a large multicentre trial in patients with early breast cancer, which recruited 4124 women from 103 participating UK hospitals, and 38 from 1 Belgian centre between January 2001 and June 2003, and compares an anthracycline–taxane chemotherapy sequence with standard UK anthracycline chemotherapy. Treatment was scheduled to last approximately 6 months. Long term follow-up will allow comparison of the rates of disease relapse and death between treatment groups. A survey of the UK patients was conducted in the autumn of 2004, well before clinical outcome data was known. Median time since randomisation was 28 months (range=15–44 months), and 282 (7%) had died. The timing aimed to capture patients' views after treatment was completed and normal day to day activities were resumed, at a time when the rate of disease relapse and death remained low and ahead of the attainment of the trial's results.

## AIM

We aimed to find out from trial patients whether they wanted to receive trial results written in lay terms when they are available, and how they considered they wanted to receive them. We compare their preferences with those expressed by health care professionals (oncologists and nurses) who had participated in the TACT trial.

## METHOD

Following ethics approval from the South East Multi-Centre Research Ethic Committee, a patient newsletter accompanied by a patient questionnaire was sent to UK hospitals to distribute to surviving TACT patients. The newsletter aimed to remind patients that follow-up continued, and explained why the trial had so far not produced any published results. Health Care Professionals (HCPs) either posted these directly to trial patients, or distributed them in the hospital clinic. Health care professionals could withhold the newsletter and questionnaire from individuals or groups of patients if they considered them inappropriate for example, those receiving palliative care. The exact number of questionnaires distributed is not known, however feedback from hospitals following an earlier patient newsletter suggests approximately 3000 of a possible 3842 were distributed.

**Figure 1:**
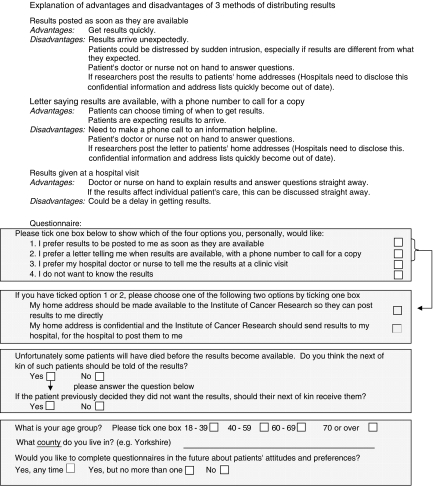
Patient questionnaire.

**Figure 2:**
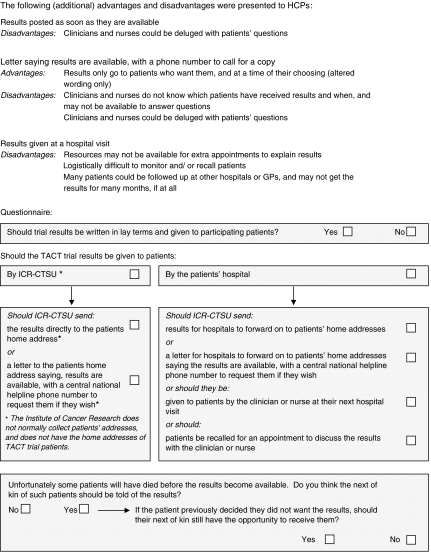
Health Care Professional Questionnaire.

The questionnaire described three methods of distributing results, and explained the advantages and disadvantages of each, as perceived by the researchers at the Clinical Trials & Statistics Unit at the Institute of Cancer Research (ICR-CTSU). Patients were then asked if they wanted results written in lay terms, and if so, which of the three methods they preferred (Figure 1). Patients were also asked about the theoretical scenario of whether and how to convey results to the next of kin of patients who had died. Information on patient age group and geographical region was collected to allow any age or regional trends to be identified. A similar survey of participating HCPs conducted simultaneously asked whether and how results should be distributed to patients, and offered the same three options. (Figure 2). Additional advantages and disadvantages, which were of no relevance to patients, were included for HCPs, which highlighted the impact of the different distribution methods on working practices. All respondents who preferred patients to receive results by post were also asked if, in principal, they thought ICR-CTSU as the coordinating trials office, should in future hold addresses so they could send results directly to patients' homes. HCPs were encouraged to circulate the questionnaire to all appropriate staff directly involved in the TACT trial, therefore the precise number distributed is not known.

## RESULTS

### Providing results to patients

#### Patients' views

In all, 37% (1431) of the UK TACT trial population who remained alive at the time the questionnaire was distributed completed and returned it (Table 1). Of those who responded, 30 (2%) patients stated that they did not want trial results, and 6 (<1%) did not answer this question (Table 2). A total of 612 patients (44% of the 1395 who wanted results) thought that for future trials, communication of results to patients should come directly from ICR-CTSU. There was no significant difference in age distribution (*P*=0.35) nor in current country of residence (*P*=0.5) between those who would like to have the trial results and those who do not (data not shown). No association was found between preferred method of delivery and age group (*P*=0.34), nor between preferred method of delivery and UK country of residence (*P*=0.55). The distribution between age groups and UK country is shown in Table 1.

#### Health care professionals' views

In all 176 HCPs responses came from 89 (86%) participating UK centres, of which 93 (53%) were nurses, 80 (45%) clinicians, and three (2%) did not specify (Table 2). Among HCPs there was a clear preference for communicating the results in the clinic (41%), with no difference between clinicians and nurses (*P*=0.18). as to which of the three methods of communicating results they preferred; however, the difference in response between HCPs and patients is significant (*P*<0.001).

Despite knowing that patient addresses are not held by ICR-CTSU, 28 (16%) thought that communication of results to patients should come directly from ICR-CTSU (Table 2).

### Providing results to next of kin of deceased patients

**Figure 3:**
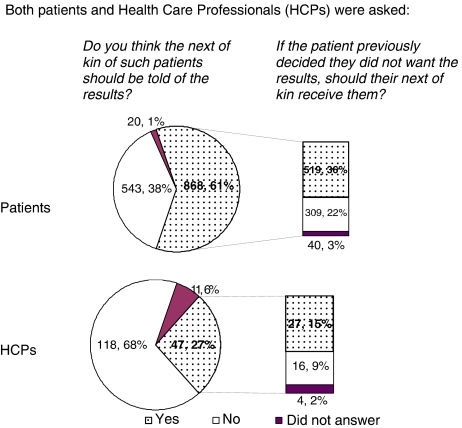
Distributing results to next of kin of deceased patients.

A total of 61% (868) patients thought that the next of kin of patients who had died should receive trial results, compared with only 27% (47) HCPs. However, of those who held this view, the proportion who also thought the results should be conveyed to the next of kin of patients who had not wanted the results was very similar for patients and HCPs (60 and 57% respectively) (Figure 3).

## DISCUSSION

Although the response rate of 37% was higher than anticipated, we cannot assume the results are representative of the views of 2411 patients who were not given the questionnaire or chose not to return it. For those who did not reply, we do not know how many did not want results, or did not feel strongly enough to complete and return it. That 30 patients (2% of respondents) felt strongly enough about not receiving the results to return the completed questionnaire highlights the need to ask patients if they want results prior to them being distributed.

Unlike trials testing ongoing treatment for a chronic disease, results of most trials of adjuvant cancer treatment have no impact on future care of participants, nor do they provide information about the future implications of trial treatment for individuals. It is commonplace within adjuvant cancer trials for the collection of long-term follow-up data to continue and the dissemination of trial results to patients could (hypothetically) introduce bias and jeopardise future knowledge and long term outcome data, particularly long-term data on quality of life. This risk, however small, needs to be balanced against the knowledge that the broad scientific trial results will only provide information about an average treatment effect of the experimental *vs* control treatment in one single trial, which should be viewed in the context of the worldwide evidence.

It is difficult for trial participants to foresee how they will react to receiving the results. For example, in a trial showing a small difference between two treatment arms, approximately 50% of patients will have received treatment, which was, on average, ‘inferior’. However, it may not be inferior for most patients who received it. In addition, highlighting the importance of clear patient information at trial entry about the uncertainty of treatment superiority, raises the question of how to explain to patients that ‘inferior’ in the trial does not necessarily mean ‘inferior’ for them, personally.

A single trial is unlikely to provide a definitive answer to the original research question; for patients in the TACT trial an unbiased account of the results would require researchers to explain them within the context of a systematic overview of the emerging worldwide data on taxanes. Added to that are the uncertainties of confidence intervals and the caveat that any promising subgroup analyses are hypothesis generating, not results in themselves. Thus trial results written in lay terms will not only fail to provide the personalised interpretation that patients may want, but if delivered without due consideration to the timing of any relapse a patient may have experienced, or without consideration of the method of distribution, there is a risk they could unnecessary heighten concerns about long-term prognosis and future clinical care.

To avoid unnecessary distress, information that accompanied this survey did not explain that results depended on enough patients relapsing or dying, yet it is this that allows statistically reliable and precise comparisons between treatment groups. Without this knowledge, can patients know whether they would want the results if they had relapsed? Patient response to receiving results could be further complicated by knowing they had also received the ‘inferior’ treatment. The timing of this questionnaire was such that very few patients had relapsed. If those few patients were excluded from receiving the questionnaire, the views of patients who have relapsed may be under-represented.

Patients were very divided on whether the next of kin of deceased patients should be given trial results, and HCPs erred towards thinking next of kin should not receive results. Qualifying comments made on questionnaires suggest this was a difficult ethical question.

The majority of patients opting to receive results by post expressed a preference for ICR-CTSU to collect patients' addresses for future trials, bypassing the hospital to convey results to patients as soon as they are available; an option that suggests a higher priority for speed than confidentiality of personal data. The responses from HCPs suggest an expectation that trial results need to be interpreted for individual patients. The lower priority given to alacrity could also suggest an awareness that peer-reviewed journals do not allow widespread dissemination of results prior to publication. In addition, results of high profile trials often fall under the media spotlight ahead of any adequate peer review. Dissemination of results by the media and the ‘spin’ put on them in the popular press may be misinterpreted by trial participants, with HCPs left to interpret results in a way that seems to patients to be less attractive.

## Figures and Tables

**Table 1:** Distribution of age groups and UK country of patients responding to questionnaires

**UK country**	**Number (%) of patients**	**Age group**	**Number (%) of patients**
England	1140 (80)	18–39	106 (7)
Scotland	102 (7)	40–59	1090 (76)
Wales	92 (6)	60–69	217 (15)
N Ireland	28 (2)	70+	15 (1)
Not specified	69 (5)		3 (<1)
Total	1431 (100)		1431 (100)

**Table 2:** Patient and HCP preferences for distributing results to patients

	**Patients (%)**	**HCPs (%)**
**Trial results**	**(*n*=1431)**	**(*n***=**176)**
Want results in lay terms (for patients)	1395 (97)	169 (96)
Do not want (patients to receive) results	30 (2)	5 (3)
Did not answer	6 (<1)	2 (1)
		

	**Patients (%)**	**HCPs (%)**
**Method of distribution preferred**	**(*n*=1395)**	**(*n*=169)**
		
*Posted to patients when available*	664 (47)	32 (19)
By patient's hospital	140 (21)	20 (62)
By ICR-CTSU	521 (79)	12 (38)
Did not answer	3 (<1)	—
		
*Letter to say results are available, with national helpline number to request a copy*	177 (13)	64 (38)
By patient's hospital	86 (49)	48 (75)
By ICR-CTSU	91 (51)	16 (25)
		
*Results given to patients by clinician or nurse*	554 (40)	70 (41)
At next hospital visit	*—*	65 (93)
Patients recalled for appointment to discuss results	*—*	5 (7)
		
Did not express any preference	*—*	3 (2)
